# Characterisation of *Escherichia coli* isolates from the blood of haematological adult patients with bacteraemia: translocation from gut to blood requires the cooperation of multiple virulence factors

**DOI:** 10.1007/s10096-015-2331-z

**Published:** 2015-02-06

**Authors:** B. Krawczyk, A. Śledzińska, K. Szemiako, A. Samet, B. Nowicki, J. Kur

**Affiliations:** 1 Department of Molecular Biotechnology and Microbiology, Faculty of Chemistry, Gdańsk University of Technology, Gdańsk, Poland; 2 Department of Therapy Monitoring and Pharmacogenetics, Medical University of Gdansk, Gdansk, Poland; 3 Laboratory of Clinical Microbiology, Gdańsk University of Medicine, Gdańsk, Poland; 4 Department of Obstetrics and Gynecology and Department of Microbiology and Immunology, Meharry Medical College, Nashville, TN USA

## Abstract

The aim of the study was to investigate whether there are unique pathotypes of *Escherichia coli* capable of transmission from the gastrointestinal tract to the vascular bed. The study included *E. coli* strains isolated from clinical materials collected from 115 patients suffering from haematologic malignancies diagnosed with bacteraemia. The genotyping techniques established that 89 *E. coli* isolates from the blood had the same genotype as the *E. coli* from the patient’s bowel. The presence of 21 genes encoding virulence factors typical of various *E. coli* pathotypes and their relationship with the phylogenetic group was established. One-dimensional analysis showed that the *focG* gene occurred more frequently in the control bowel group, while the ampicillin-resistant *afa*/*dr E. coli* were associated with bacteraemia. Blood isolates with the highest occurrence of virulence factors belonged to pathogenic group B2 and non-pathogenic group A. The co-occurrence of multiple genes encoding *papC*, *sfa*, *usp* and *cnf1* virulence factors probably predisposes *E. coli* to translocation from the gastrointestinal tract to the vascular bed in the group of patients with haematologic malignancies. Based on clustering analysis, dominance of the most virulent strains assigned to the cluster with seven virulence factors encoded by the following genes, *papC*, *sfaD*/*E*, *cnf1*, *usp*, *agn43*, *hlyA* and *iutA*, was found. The obtained results enforce the previously proposed concept of bowel–blood translocation and further expand our hypothesis by defining the unique virulence characteristics of *E. coli* isolates, which predispose them to bowel colonisation or translocation and bacteraemia in this group of patients.

## Introduction

Patients with proliferative disorders of the haematopoietic system are at a higher risk of developing bacteraemia and septicaemia [1–6]. Although the underlying mechanisms of bacteraemia are under-investigated, it is considered that both the primary disease and the treatment may contribute to the bacteraemia. The source of dissemination of bacteria is usually an extra-intestinal infection, such as respiratory, urinary tract or wound [2]. This is particularly observed in patients after the transplant of haematopoietic stem cells, when immunity is severely impaired and/or following cytostatic and immunosuppressive treatment. Research conducted in this group of patients has shown a growing number of bloodstream infections with Gram-negative rods, where *Escherichia coli* was the dominant aetiologic factor for bacteraemia and septicaemia. Mortality in patients with documented *E. coli* bacteraemia is high; depending on the research centre, it may reach even 37 % [3–5, 7–9].

We have recently reported a previously unrecognised mechanism of recurrent bacteraemia in patients with leukaemic malignancy [10]. Our study indicated that, in 70 % of patients, the bowel was the primary source of the pathogen which led to a new bacteraemic episode. The identical DNA fingerprints of *E. coli* from the bowel and blood in the absence another source of infection was consistent with interpretation that the recurrent bacteraemia occurred due to direct translocation of *E. coli* present in the bowel to the blood system.

It is considered that patients with haematologic malignancies display a varying degree of immune and intestinal barrier dysfunction [11–13]. Therefore, the significant question that remains to be addressed is whether the *E. coli* bowel to blood translocation in patients with malignant diseases occurs with a random commensal/coloniser, consistent with immune/intestine dysfunction, or with *E. coli* that carry specific virulence factors. In this instance, virulence factors would facilitate *E. coli* bowel mucosa translocation and invasion to the blood system. This question is highly relevant because the generated knowledge would allow us to design specific preventive strategies and reduce patient mortality due to *E. coli* bacteraemia.

*E. coli* pathogenesis is mediated by a broad spectrum of virulence factors specific to enteric/gastrointestinal/diarrhoeal pathogens (enterotoxins) or to extraintestinal *E. coli* (ExPEC) pathogens [14–17]. Only 10–20 % of healthy people carry ExPEC strains that stably colonise the host’s intestines [18]. ExPEC virulence is mediated by adhesins, invasins, toxins, polysaccharide coatings, siderophores and other factors targeting immune cells in the blood. Such virulence factors help to colonise the surfaces of host cells, avoid and/or abolish the host defence mechanism, damage and/or enter host cells or tissues, including blood components, and provoke a harmful immune response, which increases the risk of a disease.

The present study was conducted on a previously untested patient population. *E. coli* isolates from the blood and stool of patients with malignant diseases of the blood system were tested by DNA fingerprinting combined with a polymerase chain reaction (PCR) analysis of *E. coli* virulence factors. The obtained results enforce the previously proposed concept of bowel–blood translocation and further expand our hypothesis by defining the unique virulence characteristics of *E. coli* isolates which predispose them to bowel colonisation or translocation and bacteraemia in this group of patients.

## Materials and methods

### Bacterial strains and microbiological procedures

The study involved 115 patients treated for haematologic malignancies in the Department of Haematology and Transplantation at the University Clinical Center, in the years 2006–2012. The clinical analysis was retrospective in nature, and all clinical data were obtained from medical records. The median age of the patients was 49.8 years (range; 19–84 years) and 62 were male. The most common underlying haematologic malignancy was acute myelogenous leukaemia, observed in 59 patients. Eighteen patients underwent lymphocytic leukaemia, 11 non-Hodgkin’s lymphomas and 17 multiple myeloma. The basis for the selection of *E. coli* blood isolates for the investigation were clinically and microbiologically confirmed bacteraemia with *E. coli* aetiology, isolation of bacteria from blood samples, with the simultaneous isolation of the microorganism from the stool or anal swab. Patients were selected for which the result of the urine culture was negative [<1,000 colony-forming units (CFU)/mL] and for which there was no alternative primary site of infection. Out of the 115 patients with bacteraemia gathered, the collection included a total of 1,411 *E. coli* isolates: on average, each patient is represented by one blood isolate and up to ten bowel *E. coli* isolates. In the design of the study, we adopted the following principle: after taking into account differences in the macroscopic assessment of bacterial colony morphology, only different *E. coli* isolates were included, usually one blood and three or more different bowel isolates. As described below, *E. coli* isolates from patients with bacteraemia were genotyped and divided into two study groups: group 1, *E. coli* isolates from the blood, which had a counterpart isolate in the bowel that was identical to the blood DNA fingerprint (blood+/bowel+), and control group 2 comprising the remaining bowel isolates with DNA fingerprints that were different to those in blood and represented *E. coli* isolates which persisted in the bowel but, presumably, were not able to translocate from the bowel to blood (blood−/bowel+).

The study of the biochemical characteristics for the 1,411 isolates of *E. coli* were performed on the basis of 42 freeze-dried substrates in panel MicroScan® Rapid Negative ID Type 4, read with the help of automated WalkAway SI used for species identification and susceptibility testing of microorganisms, using microanalysis technology for the rapid identification of fluorochromes (MicroScan Rapid Fluorogenic Identification). In all cases, the correctness of *E. coli* identification was 99.99 %.

### Genotyping by PCR MP and PFGE methods

Genomic DNA was isolated from individual bacterial colonies using a commercial kit (Genomic DNA Kit, ISOLATE II Bioline). The polymerase chain reaction melting profiles (PCR MP) procedure was carried out according to the method described for *E. coli* isolates by Krawczyk et al. [19]. Relationships between strains from blood and the gastrointestinal tract were also confirmed by studying the pulsed-field electrophoresis (PFGE) patterns of genomic DNA after restriction by *Xba*I according the CHEF Mapper® XA Bio-Rad manual protocol.

### Determination of *E. coli* phylogenetic group (phylogroup)

The membership to a phylogenetic group of each *E. coli* strain was studied according to Clermont et al.’s method based on multiplex PCR using three pairs of primers [20].

### PCR screening of virulence genes

The three multiplex PCRs were validated for 12 virulence genes as a home-made test and one commercial multiplex PCR test for seven virulence genes. Next to this, two genes were separately amplified as a simplex. In cases of doubt, the multiplex PCR experiments were repeated in a simplex PCR system. Details of the primer sequences and predicted sizes of the amplified products have been described previously [14–16, 21–29]. Primers for each virulence factor were first validated individually by the use of template DNA from appropriate positive and negative control strains. The virulence factors characteristic mainly for the uropathogenic strains (UPEC) were included in multiplex PCR system I: P fimbriae assembly outer membrane usher (*papC* gene), S fimbriae (*sfaD*/*E* gene), type 1 fimbriae (*fimG*/*H* gene), cytotoxic necrotising factor 1 (*cnf1* gene), uropathogen-specific protein Usp (*usp* gene), haemolysin toxin protein (*hlyA* gene). Reactions were carried out according to Adamus-Białek et al. [21]. In a separate PCR assay, the presence of Dr fimbriae (*draC–D*) was tested [27].

In multiplex PCR system II, the sequence of three fragment genes, *kspMTII* [23] (synthesis capsule, group II), *iha* [28] (enterobactin Iha-iron-regulated gene homologue adhesion) and *focG* [24, 28] (F1C fimbriae), were amplified. In multiplex PCR system III, the sequence of three fragment genes, *iutA* [25] (aerobactin receptor), *fyuA* [26] (yersiniabactin receptor) and *ibeA* [23, 24] (invasion of brain endothelium A), were amplified.

For the detection of virulence genes typical for diarrhoeagenic *E. coli* (DEC), the commercial DEC PCR Kit (Statens Serum Institute, Denmark) was used. In this multiplex PCR system IV, the detection of *vtx1* and *vtx2* (verocytotoxin strains, VTEC), *eae* (EPEC strains), *eltA*, *estA*-*human* or *estA*-*porcine* (ETEC strains), and *ipaH* (EIEC strains) were included. An *agn43* gene was detected by simplex PCR according to Kotlowski et al. [29].

### Statistical analysis

Data were statistically analysed using the Microsoft Excel 2007 program. Statistical testing was done using the SPSS Statistics 19 program. In order to select the characteristics of *E. coli* strains that predispose to translocation from the gastrointestinal tract into the blood, we performed a Chi-square analysis. *p*-Values less than 0.05 were considered to be significant. Cluster analysis was performed using the k-means method (QUICK CLUSTER).

## Results

### Genotyping

The study group included a collection of *E. coli* isolates obtained from clinical materials from a group of 115 adult patients with blood malignancy with microbiologically and clinically documented *E. coli* bacteraemia. On the basis of the genotyping results, *E. coli* isolates were divided into two groups: group 1, 89 *E. coli* isolates from the blood which had a counterpart isolate in the bowel that was identical to the blood DNA fingerprint (blood+/bowel+) and control group 2 comprising the remaining 146 *E. coli* bowel isolates with DNA fingerprints that were different to those in blood and represented *E. coli* isolates in the bowel but absent in blood: presumably, *E. coli* isolates of group 2 were not able to translocate from the bowel to blood (blood−/bowel+). The remaining 50 isolates from blood, for which the genotypes were not found among isolates from the bowel (blood+/bowel−), were excluded from this evaluation. We consider that these 50 isolates originated either from an unknown extraintestinal source or were underrepresented among the bowel isolates. Strains of blood+/bowel+ and blood−/bowel+ as a control group were further analysed.

### Prevalence of virulence factor genes

The unidirectional analysis of PCR-based detection of genes encoding the examined *E. coli* virulence factors (Table 1) indicates that only the *afa*/*dr* region predominated in blood isolates; 16 % of strains from blood vs. 5 % in the control bowel group (*p* = 0.009). In contrast, the *focG* gene (encoding F1C fimbriae) was more often identified among control bowel colonisers (*p*-value = 0.001); 36 % of the control blood−/bowel+ group strains vs. 17 % of the blood+/bowel+ isolates; note that all blood isolates with corresponding colon isolates displayed identical virulence patterns.

**Table 1:** Unidirectional analysis of polymerase chain reaction (PCR)-based detection of genes encoding the examined *Escherichia coli* virulence factors

Virulence factor^a^	Blood+/bowel+ group (*n* = 89)			Blood−/bowel+ group (*n* = 146)			*p*-Value
	*n*	%^b^	%^c^	*n*	%^b^	%^c^	
*afa*/*dr*	14	16	64	8	5	36	**0.009**
*fimG*/*H*	59	66	39	91	62	61	0.540
*sfa*	47	53	42	66	45	58	0.258
*papC*	45	51	44	57	39	56	0.084
*hlyA*	28	31	42	38	26	58	0.369
*usp*	43	48	36	76	52	64	0.578
*cnf1*	44	49	39	69	47	61	0.746
*fyuA*	57	64	38	92	63	62	0.874
*iutA*	67	75	39	104	71	61	0.499
*ibeA*	26	29	30	61	42	70	0.053
*iha*	37	42	41	53	36	59	0.420
*focG*	15	17	22	53	36	78	**0.001**
*kspMTII*	33	37	43	44	30	57	0.271
*agn43*	72	81	40	107	73	60	0.184

^a^ *afa*/*dr* Dr fimbriae (*afa*/*draB–C*); *fimG*/*H* type 1 fimbriae (*fimG*/*fimH*); *sfa* S fimbriae (*sfaD*/*sfaE*); *papC* P fimbriae; *hlyA* haemolysin; *usp*bacteriocin Usp; *cnf1* cytotoxic necrotising factor; *fyuA* yersiniabactin receptor; *iutA* aerobactin receptor; *ibeA* invasive protein; *iha* enterobactin (siderophore receptor and adherence factor); *focG* F1C fimbriae; *kspMTII* protein responsible for capsule formation; *agn43* adhesin 43 (biofilm formation)

^b^Percent within the group of isolates

^c^Percent within the virulence factor

No genes encoding virulence factors characteristic of DEC were detected in the PCR reactions in tested blood isolates (data not shown). The lack of these virulence factors was also confirmed by serological tests conducted (data not shown). An intriguing possibility exists that F1C fimbriae could enhance bowel colonisation, while invasion coding *afa*/*dr* could contribute to translocation to the bloodstream.

### Multidimensional analysis of the occurrence of virulence factors (co-occurrence)

As our previous report [30] indicated that the translocation to blood may be increased by the presence of Dr adhesins together with P fimbriae in patients with urosepsis, we evaluated the relationship between multiple virulence factors and translocation in patients with haematologic malignancies. As shown in Table 2, the following pairs of genes, *hlyA* + *iutA*, *agn43* + *papC*, *focG* + *fimG*, *kspMTII* + *agn43*, *cnf1* + *papC*, *cnf1* + *sfa*, *usp* + *papC*, *usp* + *sfa*, *iutA* + *agn43*, were present at a higher frequency among blood+/bowel+ isolates, in particular, the *cnf1* + *papC* and *usp* + *papC* pairs, for which the *p*-values were 0.009 and 0.001, respectively. The co-occurrence of *ibeA* + *iutA* genes was more frequently observed for isolates from the control blood−/bowel+ group.

**Table 2:** Two-dimensional analysis of the coexistence of two genes encoding virulence factors among the investigated *E. coli* isolates: statistically important results for the discrimination of two groups of strains

Virulence factor^a^	Blood+/bowel+ group (*n* = 89)		Blood−/bowel+ group (*n* = 146)		*p*-Value
	*n*	%^b^	*n*	%^b^	
*hlyA* + *iutA*	25	28	26	18	0.046
*agn43* + *papC*	39	44	41	28	0.036
*focG* + *fimG*/*H*	14	16	36	25	0.049
*ibeA* + *iutA*	20	22	57	39	0.027
*kspMTII* + *agn43*	30	34	32	22	0.046
*cnf1* + *papC*	29	33	28	19	**0.009**
*cnf1* + *sfa*	39	44	50	34	0.040
*usp* + *papC*	32	36	32	22	**0.001**
*usp* + *sfa*	35	39	48	33	0.037
*iutA* + *agn43*	58	65	74	51	0.019

^a^Virulence factor names as in the legend of Table 1

^b^Percent within the group of isolates

**Fig. 1:**
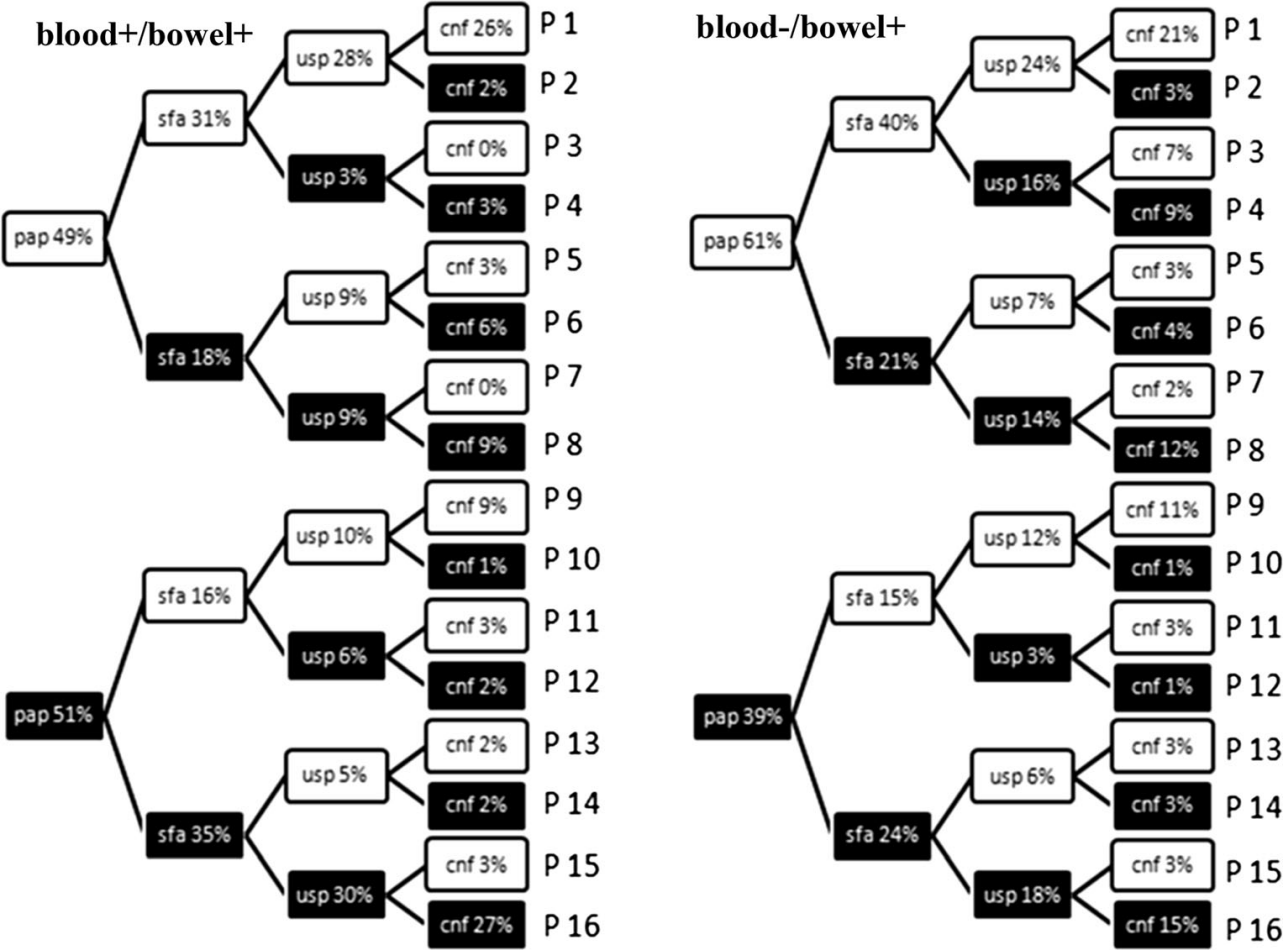
Image analysis of co-occurrence of virulence factors *papC*, *sfa*, *usp* and *cnf1* presented in the form of a dichotomic key. The *white rectangles* indicate the absence of gene encoding virulence factors and the *black rectangles* indicate the presence of gene encoding virulence factors

We then further evaluated the relationship between multiple virulence genes and translocation. Based on the co-occurrence of four virulence factors, *papC*, *sfa*, *usp* and *cnf1*, 16 profiles of co-occurrence were distinguished in each tested group of strains (Fig. 1). It was observed that profile 16 (all four factors positive) was the only one that was significantly more frequent in the blood+/bowel+ than in the blood−/bowel+ isolates groups (27 and 15 %, respectively). The co-occurrence of multiple genes encoding virulence factors, *papC*, *sfa*, *usp* and *cnf1*, among blood+/bowel+ probably predisposes *E. coli* to translocation from the gastrointestinal tract to the blood system in the group of patients with haematologic malignancies.

The two most numerous profiles, 1 and 16, were also analysed for the co-occurrence of additional virulence factors (Table 3). Statistically significant differences were shown for the co-occurrence of *fimG*/*H* and *hlyA* (*p* = 0.007 and *p* < 0.001, respectively).

**Table 3:** Summary of the percentage of co-occurrence of other virulence factors for groups blood+/bowel+ and blood−/bowel+ and profiles 1 and 16

Virulence factor^a^	Blood+/bowel+ group		Blood−/bowel+ group		*p*-Value
	Profile 1 (*n* = 23), *n*/%	Profile 16 (*n* = 24), *n*/%	Profile 1 (*n* = 31), *n*/%	Profile 16 (*n* = 22), *n*/%	
*fimG*/*H*	19/83	18/75	13/42	16/73	**0.007**
*hlyA*	1/4	14/58	1/3	18/82	**0.001**
*fyuA*	13/57	16/67	16/52	18/82	0.132
*iutA*	16/70	17/71	25/81	17/77	0.760
*ibeA*	9/39	7/29	19/61	11/50	0.100
*iha*	13/57	9/38	15/48	10/45	0.625
*focG*	4/17	5/21	13/42	9/41	0.117
*kspMTII*	7/30	12/50	11/35	8/36	0.545

^a^Virulence factor names as in the legend of Table 1

Profile 1 was characterised by a higher occurrence of the *fimG*/*H* gene among blood isolates (blood+/bowel+ group) as compared to the blood−/bowel+ group, which was treated as the control (83 and 42 %, respectively). On the other hand, profile 1 showed negligible presence of the *hlyA* gene, in contrast to profile 16 (blood+/bowel+ group, 58 %; blood−/bowel+ group, 82 %). In profile 16, the *fimG*/*H* gene occurred at an equally high level, regardless of the group (blood+/bowel+, 75 %; blood−/bowel+ group, 73 %). In contrast, the *hlyA* gene in profile 16 was less frequent in blood than in the control bowel group (blood+/bowel+ group, 58 %; blood−/bowel+ group, 82 %).

To confirm the significance of the selected virulence factors based on multidimensional analyses for strains isolated from blood, cluster analysis using the k-means method (QUICK CLUSTER) was performed. Six clusters were created for eight virulence factors (Table 4) and the number of observations was specified for each cluster. Based on clustering analysis within the specified group, dominance of the most virulent strains assigned to the cluster with seven virulence factors encoded by the following genes, *papC*, *sfaD*/*E*, *cnf1*, *usp*, *agn43*, *hlyA* and *iutA*, was found (cluster 2 in Table 4). This observation is consistent with the interpretation that dominant cluster 2 from blood+/bowel+ carried the most virulence factors and, therefore, was best equipped to translocate to the blood system. One concern remains that the analysis of profiles 1 and 16 in Fig. 1 and Table 3 suggested a surprising observation that type 1 adhesin contributed to bacteraemia while haemolysin did not. We consider that an unexpected lack of association of Hly with virulence resulted from the altered phylogenetic distribution.

**Table 4:** Cluster analysis of *E. coli* strains of the blood+/bowel+ group. Six clusters with various profiles of eight virulence factors: *papC*, *sfaD*/*E*, *usp*, *cnf1*, *agn43*, *hlyA*, *iutA* and *ibeA*

Clusters^a^	*n*	%
1	16	18
2	33	37
3	10	11
4	1	1
5	4	4
6	25	28

^a^1: no virulence factors; 2: *papC*, *sfaD*/*E*, *usp*, *cnf1*, *agn43*, *hlyA*, *iutA*; 3: *sfaD*/*E*, *usp*, *cnf1*, *agn43*; 4: *papC*, *sfaD*/*E*, *cnf1*, *ibeA*; 5: *papC*, *agn43*, *hlyA*, *iutA*, *ibeA*; 6: *agn43*, *iutA*

### Phylogenetic grouping

For isolates from the blood+/bowel+ group, four phylogenetic groups were identified: group A, 14 (16 %); group B1, 27 (30 %); group B2, 40 (45 %); and group D, 8 (9 %). The results were referred to the control group (blood−/bowel+), where affiliation to group A [30 (21 %)], group B1 [41 (34 %)], group B2 [59 (40 %)] and group D [8 (5 %)] was found. The affiliation to phylogenetic group proved to be statistically insignificant (*p*-value = 0.545). The majority of strains, regardless of the isolation source, belonged to phylogenetic group B2, which is considered to be pathogenic. A relatively large number of strains belonged to phylogenetic group B1, classified as physiological microflora in the gastrointestinal tract. We consider that group A and B1 isolates could migrate to the circulatory system in this group of patients, due to damaged intestinal mucosa caused by therapy with cytostatic medication, ionising radiation and broad-spectrum antibiotics. However, this suggestion requires further verification for the occurrence of virulence factors (see below).

At the same time, an analysis of phylogenetic affinity was performed for profiles 1 and 16 of both groups (Table 5). Regardless of the strain origin, phylogroup B1 was the most numerous for profile 1 (low frequency of virulence factors), while phylogroup B2 occurred in the greatest numbers for profile 16 (high frequency of virulence factors). In addition, for strains isolated from blood (blood+/bowel+ group) belonging to profile 16, there was a much higher percentage of strains from phylogenetic group A (29 %) as compared to profile 16 of the control group (18 %). It was shown that the four groups of strains are statistically significantly different due to the phylogenetic groups distribution (*p* = 0.033). The *p*-value was calculated for strains divided into four groups (*n* = 100) and phylogenetic groups (rows) (Table 5).

**Table 5:** Summary of the percentage of phylogenetic groups for groups blood+/bowel+ and blood−/bowel+ and profiles 1 and 16

Phylogenetic group	Blood+/bowel+ group		Blood−/bowel+ group	
	Profile 1 (*n* = 23), *n*/%	Profile 16 (*n* = 24), *n*/%	Profile 1 (*n* = 31), *n*/%	Profile 16 (*n* = 22), *n*/%
A	3/13	7/29	6/19	4/18
B1	11/48	3/13	15/48	4/18
B2	7/30	11/46	9/29	14/64
D	2/9	3/13	1/3	0/0

### Virulence factors and the phylogenetic group

Table 6 presents the analysis of the virulence factor profiles among the phylogenetic groups of *E. coli* strains isolated from blood (blood+/bowel+ group). Consistent with the predicted virulence of group B2, the occurrence of the *sfa*, *hlyA*, *cnf1* and *fyuA* genes (*p* = 0.010; *p* < 0.001, *p* = 0.009 and *p* = 0.013, respectively) was statistically significant in phylogenetic group B2. It was observed that no *focG* and *hlyA* genes occurred in strains belonging to phylogenetic group D. Interestingly, commensal group A showed a high frequency of several virulence genes as *fimG*/*H*, *papC*, *sfa* and *usp*, while B1 showed a very low frequency of *hlyA*. When the results obtained for the blood+/bowel+ group of strains are compared with the data obtained for the control blood−/bowel+ group (Table 7), it can be observed that virulence factors were distributed among phylogenetic groups evenly, with no statistically dominant virulence factors. An exception was the *ibeA* gene characteristic of isolates from the control group of phylogenetic group B1 (53 %, *p*-value = 0.049). Overall, these observations support the explanation that, in patients with haematologic malignancies, virulence factors appear to spread among commensal *E. coli* within the patient bowel.

**Table 6:** Analysis of the virulence factor profiles among the phylogenetic groups of *E. coli* strains of the blood+/bowel+ group

Virulence factor^a^	Phylogenetic group												*p*-Value
	A (*n* = 14)			B1 (*n* = 27)			B2 (*n* = 40)			D (*n* = 8)			
	*n*	%^b^	%^c^	*n*	%^b^	%^c^	*n*	%^b^	%^c^	*n*	%^b^	%^c^	
*afa*/*dr*	3	21	21	6	43	22	4	29	10	1	7	13	0.521
*fim*	13	22	93	17	29	63	242	41	60	5	8	63	0.150
*sfa*	9	19	**64**	7	15	**26**	26	55	**65**	5	11	**63**	**0.010**
*papG*	10	22	71	9	20	33	22	49	55	4	9	50	0.114
*hlyA*	7	25	**50**	2	7	7	19	68	**48**	0	0	0	**0.000**
*usp*	9	21	64	11	26	41	19	44	48	4	9	50	0.558
*cnf*	8	18	**57**	6	14	**22**	25	57	**63**	5	11	**63**	**0.009**
*fyuA*	7	12	**50**	13	23	**48**	33	58	**83**	4	7	**50**	**0.013**
*iutA*	9	13	64	19	28	70	32	48	80	7	10	88	0.497
*ibeA*	4	15	29	8	31	30	11	42	28	3	12	38	0.955
*iha*	6	16	43	8	22	30	20	54	50	3	8	38	0.420
*focG*	3	20	21	5	33	19	7	47	18	0	0	0	0.594
*kspMTII*	4	12	29	8	24	30	17	52	43	4	12	50	0.541
*agn43*	10	14	71	19	26	70	35	49	88	8	11	100	0.124

^a^Virulence factor names as in the legend of Table 1

^b^Percent within the virulence factor

^c^Percent within the phylogenetic group

**Table 7:** Analysis of the virulence factor profiles among the phylogenetic groups of *E. coli* strains isolated from blood (*n* = 89) and the gastrointestinal tract (*n* = 146)

Virulence factor^a^	Phylogenetic group							
	A (^b^ *n* = 14/30)		B1 (^b^ *n* = 27/49)		B2 (^b^ *n* = 40/59)		D (^b^ *n* = 8/8)	
	*n*	%^c^	*n*	%^c^	*n*	%^c^	*n*	%^c^
*afa*/*dr*	3/1	21/3	6/2	22/4	4/5	10/8	1/0	13/0
*fim*	13/22	93/73	17/26	63/53	24/39	60/66	5/4	63/50
*sfa*	9/6	^d^ **64**/20	7/19	26/39	26/37	65/63	5/4	63/50
*papG*	10/13	71/43	9/16	33/33	22/27	55/46	4/1	50/13
*hlyA*	7/7	50/23	2/12	7/24	19/18	48/31	0/1	0/13
*usp*	9/19	64/63	11/21	41/43	19/32	48/54	4/4	50/50
*cnf*	8/11	57/37	6/20	22/41	25/35	63/59	5/3	63/38
*fyuA*	7/17	50/57	13/30	48/61	33/41	83/69	4/4	50/50
*iutA*	9/18	64/60	19/36	70/73	32/44	80/75	7/6	88/75
*ibeA*	4/14	29/47	8/26	30/^d^ **53**	11/20	28/34	3/1	38/13
*iha*	6/10	43/33	8/16	30/33	20/24	50/41	3/3	38/38
*focG*	3/6	21/20	5/19	19/39	7/27	18/^d^ **46**	0/1	0/13
*kspMTII*	4/5	29/17	8/14	30/29	17/22	43/37	4/3	50/38
*agn43*	10/17	71/57	19/37	70/76	35/48	88/81	8/5	100/63

^a^Virulence factor names as in the legend of Table 1

^b^Number of isolates in the phylogenetic group [blood+/bowel+]/[blood−/bowel+]

^c^Percent within the phylogenetic group

^d^ *p*-Value <0.005

### Drug resistance profile

An assessment of the antibiotic resistance profile was performed for pairs of strains from the blood and the gastrointestinal tract (*n* = 89) in the sensitive/resistant categories and minimum inhibitory concentration (MIC) values testing. On the basis of the results obtained, it can be concluded that the tested strains were sensitive to the majority of the selected antibiotics. For 11 antibiotics (piperacillin, piperacillin with tazobactam, cefuroxime, cefotaxime, ceftazidime, cefepime, imipenem, ertapenem, amikacin, gentamicin and netilmicin), over 90 % of the tested strains were found to be sensitive. The largest percentage of resistant strains was observed for ampicillin (44 %), ciprofloxacin and norfloxacin (35 % each). It was interesting that all 22 strains with the *afa*/*dr* virulence factor gene isolated from both groups were ampicillin resistant. Due to the low frequency of multidrug-resistant strains among the tested isolates, it can be inferred that these are not strains of hospital origin. On the other hand, the association of Dr adhesins with resistance to ampicillin appears to be an independent risk factor for bacteraemia.

## Discussion

Here, we present findings which re-enforce the recently proposed concept that, in patients with haematologic malignancies, the bowel is a main source of direct *E. coli* bowel–blood translocation and resulting bacteraemia [10]. We further expand our hypothesis by defining the virulence characteristics of *E. coli* bowel–blood colonisers and provided evidence that bowel colonisation and risk of translocation and bacteraemia in this group of patients was associated with *E. coli* bearing genes encoding specific sets of colonisation/invasion/toxic factors.

Genotyping analysis of *E. coli* among 115 patients suffering from haematologic malignancies, in whom bacteraemia was diagnosed, was performed. *E. coli* isolates with the same genotype in the blood and the bowel were found in the majority of patients and were not detected in only 31 patients. 64 % (*n* = 89) of isolates from the blood found their counterparts in the bowel (blood+/bowel+). The findings are consistent with our previous report that, in patients with haematologic malignancies, in the absence of extraintestinal and gastrointestinal source of infection, the bowel remains the most likely source of *E. coli* direct translocation to the bloodstream [10]. We further investigated an unresolved question regarding whether *E. coli* bowel to blood translocation in patients with malignant diseases occurs with a random commensal/coloniser or with *E. coli* that carry specific virulence factors. Our data support the hypothesis that unique virulence genes of *E. coli* isolates predispose them to translocation and bacteraemia, while different factors contribute to bowel colonisation in this group of patients.

The results of one-dimensional analysis were statistically significant for the *afa*/*dr* gene, which was associated with strains of blood+/bowel+ group, and the *focG* gene, which occurred more frequently in the control blood−/bowel+ group. *E. coli* bearing *afa*/*dr* genes coding Dr and Afa adhesins originate from the gastrointestinal tract as the host’s intestinal microflora [31], mediate tissue invasion and can cause chronic urinary tract infections and bacteraemia [30–34]. The present observation suggests that Dr adhesins can represent an independent factor predisposing for bacteraemia in the tested patients. In this view, the question arises as to whether the presence of virulent strains with Dr/Afa-type adhesins in the gastrointestinal tract of patients with malignancies and immunosuppression pose a risk of bacteraemia and require specific eradication strategy. An intriguing possibility exists that F1C fimbriae coded by the *focG* gene could enhance bowel colonisation and, perhaps, restrict *E. coli* translocation to the bloodstream. This is in accordance with recent observations that F1C fimbriae contributed to *E. coli* persistence in an infant mouse intestinal colonisation and biofilm formation model [35].

As our previous report indicated, in patients with urosepsis, the bowel–blood translocation may be increased by the presence of two adhesins, Dr and P fimbriae. We evaluated the relationship between pairs, clusters of four and clusters of more than four most frequent virulence factors and blood–bowel translocation in patients with leukaemia [30]. This analysis showed pairs of genes which differentiated both groups of strains; the pairs of genes were *hlyA* + *iutA*, *agn43* + *papC*, *focG* + *fimG*, *kspMTII* + *agn43*, *cnf1* + *papC*, *cnf1* + *sfa*, *usp* + *papC*, *usp* + *sfa*, *iutA* + *agn43*. The most frequent profiles of four virulence factors were *papC*, *sfa*, *usp* and *cnf1*. Profile 16 (all four factors positive) and profile 1 (all four factors negative) constituted more than half of all combinations within the blood+/bowel+ group. When the two most numerous profiles 1 and 16 were analysed for the co-occurrence of other factors, the association of type 1 fimbriae but not haemolysin with bacteraemia was noted. We consider that an unexpected lack of association of *hlyA* with bacteremia resulted from altered phylogenetic distribution. The multidimensional clustering analysis confirmed the dominance of the most virulent strains assigned to the cluster 2, with seven virulence factors encoded by the following genes, *papC*, *sfaD*/*E*, *cnf1*, *usp*, *agn43*, *hlyA*, *iutA*, in bacteraemia. This observation is consistent with the interpretation that dominant cluster 2 from the blood+/bowel+ group carried most virulence factors, including haemolysin, and, therefore, was best equipped to translocate to the blood system. The co-occurrence of multiple factors may result in a cooperative virulence strategy [30]. P fimbriae have the ability to bind to epithelial cells and signal inflammatory response, and the Usp bacteriocin has exonucleolytic properties and the cytotoxic necrotising factor encoded by *cnf1* can cause cell injury, all of which are rarely encountered in commensal strains. We consider that, in patients with haematologic malignancies, these bacterial factors, in combination with the host responses, may result in epithelial cells dysfunction, which become more permeable for microorganisms invasion, allowing their translocation/transmission.

The correlation of virulent genotypes and the phylogenetic background for *E. coli* strains isolated from blood samples in our material is consistent with the data presented in the literature. Phylogenetic group B2 is considered to be pathogenic and rich in various virulence factors. Our observation showed that, within the commensal phylogenetic group A, a higher occurrence of virulence factors was found in strains obtained from the blood than in strains from the gastrointestinal tract. We consider that an unexpected lack of association of *hlyA* with bacteraemia in co-occurrence with the four most frequent factors resulted from an altered phylogenetic distribution, e.g. very low or no occurrence in groups B1 and D. It was noted that the horizontal and vertical transfer of genes encoding virulence factors leads to high differentiation of the virulence degree among all phylogenetic groups [16, 30, 32, 36]. No such significant differences were noticed for the phylogenetic group B2 of strains from the blood and the control group; in both cases, a high diversity of the combination of virulence factors was detected. The question arises as to whether the growth of pathogenic microflora in patients with haematologic malignancies could lead to enhanced transfer of genes encoding virulence factors to strains from the commensal group and, hence, probably a selection of new subtypes of *E. coli* pathogens posing a risk for the general population. In a similar context, the clonal selection of *E. coli* pathogen equipped with virulence factor(s) such as Dr adhesin and antibiotic resistance, therefore capable of invading/persisting within infected cells and escaping clearance by antibiotic(s) and disseminating to blood as previously proposed by Nowickis’ group, may represent a significant epidemiologic risk in sensitive populations [37].
